# Simulated Warming Differentially Affects the Growth and Competitive Ability of *Centaurea maculosa* Populations from Home and Introduced Ranges

**DOI:** 10.1371/journal.pone.0031170

**Published:** 2012-01-30

**Authors:** Wei-Ming He, Jing-Ji Li, Pei-Hao Peng

**Affiliations:** 1 State Key Laboratory of Vegetation and Environmental Change, Institute of Botany, the Chinese Academy of Sciences, Beijing, China; 2 Department of Landscape Architecture, Chengdu University of Technology, Chengdu, China; Ohio State University, United States of America

## Abstract

Climate warming may drive invasions by exotic plants, thereby raising concerns over the risks of invasive plants. However, little is known about how climate warming influences the growth and competitive ability of exotic plants from their home and introduced ranges. We conducted a common garden experiment with an invasive plant *Centaurea maculosa* and a native plant *Poa pratensis*, in which a mixture of sand and vermiculite was used as a neutral medium, and contrasted the total biomass, competitive effects, and competitive responses of *C. maculosa* populations from Europe (home range) and North America (introduced range) under two different temperatures. The warming-induced inhibitory effects on the growth of *C. maculosa* alone were stronger in Europe than in North America. The competitive ability of *C. maculosa* plants from North America was greater than that of plants from Europe under the ambient condition whereas this competitive ability followed the opposite direction under the warming condition, suggesting that warming may enable European *C. maculosa* to be more invasive. Across two continents, warming treatment increased the competitive advantage instead of the growth advantage of *C. maculosa*, suggesting that climate warming may facilitate *C. maculosa* invasions through altering competitive outcomes between *C. maculosa* and its neighbors. Additionally, the growth response of *C. maculosa* to warming could predict its ability to avoid being suppressed by its neighbors.

## Introduction

Temperatures are one of the most fundamental factors shaping plant growth and distribution in terrestrial ecosystems [1], thus changing temperatures are likely to shift the growth and distribution of plant species. Global surface temperatures are projected to increase by 1.8–4.0°C by the end of this century [2] and invasive plants are currently expanding [3], [4], thereby raising concerns over whether climate warming increases the risks of invasive plants [4], [5]. Predicting future risks of plant invasions also requires knowledge on the performance of invaders in their home and introduced ranges [3], [6].

*Centaurea maculosa* is native to Europe and a highly notorious forb in North America. North American *C. maculosa* populations exhibit genetic differences from European counterparts due to rapid evolution [7]–[9]. Our recent research also suggests that *C. maculosa* populations may be evolving towards being a good competitor in its introduced range, but at the cost of being a good stress tolerator [10]. Thus, *C. maculosa* provides an opportunity to examine the biogeographic effects of climate warming on its invasion risks through comparing populations from its home and invaded range. Although extensive studies have been focused on the invasiveness of *C. maculosa* [10]–[14], little is known about how climate warming influences its growth and competitive ability.

Climate warming can increase the growth and production of exotic plants through physiological processes and changes in the length of growing seasons [15], [16]. Warming effects on growth are tightly associated to source-sink relationships of photosynthesis; if greater carbohydrate consumption by plant respiration can stimulate photosynthesis, then climate warming exhibits facilitation [17], [18]. In some cases, climate warming also has antagonistic effects on population growth of invasive plants through reducing seed production and emergence [19]. Evidence from model simulations has shown that rising temperatures may increase or decrease invasion risks [3].

Our central hypothesis was that identical warming may differentially affect the growth and competitive ability of *C. maculosa* populations from its home and introduced range. The evolution of increased competitive ability (EICA hypothesis) predicts that invasive plants are characterized by faster growth and higher competitive ability [20]. Due to the absence of natural enemies, introduced populations could re-allocate energy and resources to growth and thus evolve increased competitive ability. If climate warming favors this reallocation and also suppresses local plants, then novel selection pressures may allow *C. maculosa* populations from North America to exhibit greater growth and competitive advantages. Thus this study was also to test EICA hypothesis in the context of climate warming. Our secondary hypothesis was that climate warming will enhance growth and competitive advantages of *C. maculosa*. We tested these hypotheses by conducting a common garden experiment. To fully understand the competitive ability of *C. maculosa*, we quantified its effect of competition and response to competition simultaneously.

## Materials and Methods

*Centaurea maculosa* L. is native to Europe and was introduced into North America in the 1890s [6], [21]. Since then *C. maculosa* has been expanding and thus become among the most noxious weeds in the United States of America (USA) and southern Canada [21]. To compare the biogeographic effects of climate warming on the growth and competitive ability of *C. maculosa*, its seeds were collected from its home range (Austria, France, Romania, and Ukraine) and invaded range (Arkansas, Maryland, Montana, and Vermont in the USA and British Columbia in Canada). For the coordinates of sampling sites see our recent paper [10]. These collections can represent a substantial portion of the range of *C. maculosa* populations in Europe and North America, respectively.

*Poa pratensis* L. (Poaceae) is one common grass in Europe and North America. *Centaurea maculosa* and *P. pratensis* can co-occupy some habitats. If *C. maculosa* further expands its distribution under climate warming, then *P. pratensis* is most likely to become one of the most common competitors against *C. maculosa*. This situation sets up a stage for understanding interactions between *C. maculosa* and *P. pratensis* under climate warming. We chose *P. pratensis* as a target competitor to indicate the potential risks of *C. maculosa*. The seeds of *P. pratensis* were collected from Missoula, Montana in the USA.

We conducted an experiment using European and North American *C. maculosa* at our common experimental garden, and thus no specific permits were required for this study. Plants from the four European and five North American populations of *C. maculosa* were each planted in competition with *P. pratensis*, testing interspecific competition, and two plants in a container were 2–3 cm apart; plants from all *C. maculosa* populations and *P. pratensis* were also grown alone as the controls. There were 10 replicates for each treatment. All plants were grown from seeds and grown in 1500 cm^3^ containers (upper diameter: 12 cm; lower diameter: 8 cm; height: 20 cm), and all the population-competition combinations were subjected to each of the two different temperatures (i.e. ambient and warming temperatures). The warmed treatment was heated continuously during the experiment with a HS–2408 infrared radiator (Kalglo Electronics, Bethlehem, PA, USA) that was suspended 1.5 m aboveground. One ‘dummy’ heater with the same shape and size as the infrared radiator was used to simulate the shading effect of the infrared radiator in the unwarmed control. This heating approach increased the air temperatures surrounding the target plants by about 2°C (ranging between 1.5–2.5°C), which is in the magnitudes of climate warming projected by IPCC [2].

It is important to note that we used a mixture of 1∶1 sand and vermiculite (not natural soil) as a growth medium because it was neutral to all individuals and provided the same soil environment for them. In contrast, if we used a natural soil from the home or invaded range of *C. maculosa*, soil biota might yield complicated side effects [12]. We supplied each plant with adequate nutrients through fertilizing (i.e. 50 ml of a 0.5% nutrient solution, Peters Professional, 20% N, 20% P2O5, 20% K2O, Scotts Company, USA) every two weeks. The light and rainfall were identical for all plants. Water was also supplemented as required. This experiment ran from May 2009 to September 2009, during which the total rainfall and average air temperature were 350 mm and 28°C. At the end of the experiment, all plants were harvested and their total biomasses (including above- and belowground parts) were determined after oven-drying for 72 h at 60°C.

To quantify the effects of warming on the growth of *C. maculosa* populations, we calculated the change in biomass (CB) through the following equation: CB = (*B_w_*−*B_a_*)/*B_a_*×100%, where *B_w_* is the biomass of a plant grown alone but subjected to warming and *B_a_* is the biomass of plants grown alone under the ambient condition. To quantify the effects of the population origin and warming on competitive ability, we calculated relative interaction intensity (RII) through the following method: RII = (*B_c_*−*B_o_*)/(*B_c_*+*B_o_*), where *B_c_* is the biomass of a plant when growing with other plants and *B_o_* is the biomass potentially achieved by target plants growing in absence of interspecific interactions [22]. Here we separated competitive ability into two components: *competitive effect* and *competitive response*, because the competitive ability of a plant is determined by these two aspects simultaneously. *Competitive effect* indicates the potential of a target plant to suppress its neighbors and *competitive response* indicates the ability of a target plant to avoid being suppressed by its neighbors [23]. When *P. pratensis* was used as target plants, the RII values reflected the competitive effect of *C. maculosa* populations on *P. pratensis*; when *C. maculosa* alone was used as target plants, the RII values reflected the response of *C. maculosa* populations to competition due to the presence of *P. pratensis*. Thus RII values can indicate either competitive effects or competitive responses, depending on the target plant per se. RII has values ranging from −1 to 1, and is negative for competition and positive for facilitation [22]. To further quantify the effects of warming on the competitive ability of *C. maculosa* populations, the change in competitive effects and competitive responses was calculated as: (*RII_w_*−*RII_a_*)/*RII_a_*×100%, where *RII_w_* is the RII value of a plant subjected to warming and *RII_a_* is the RII value of plants grown in the ambient condition.

We used the PROC GLM module within SAS using Type III sum of squares (version 9.1), where the continent of population origin (Europe versus North America) was treated as a fixed factor, climate (ambient versus warming) was treated as a fixed factor, and population nested within a continent was treated as a random factor. We tested the effects of these factors and their interactions on total biomass, competitive effects, and competitive responses. We also used the PROC GLM module within SAS using Type III sum of squares (version 9.1), where the continent of population origin (Europe versus North America) was treated as a fixed factor and population nested within a continent was treated as a random factor. We tested the effects of the continent of population origin on the change in biomass, competitive effects, and competitive responses with warming. We used a correlation analysis (SPSS 15.0, SPSS Inc., Chicago) to explore the relationship between the change in biomass with warming and both the change in competitive effects with warming and the change in competitive responses with warming.

## Results

**Figure 1:**
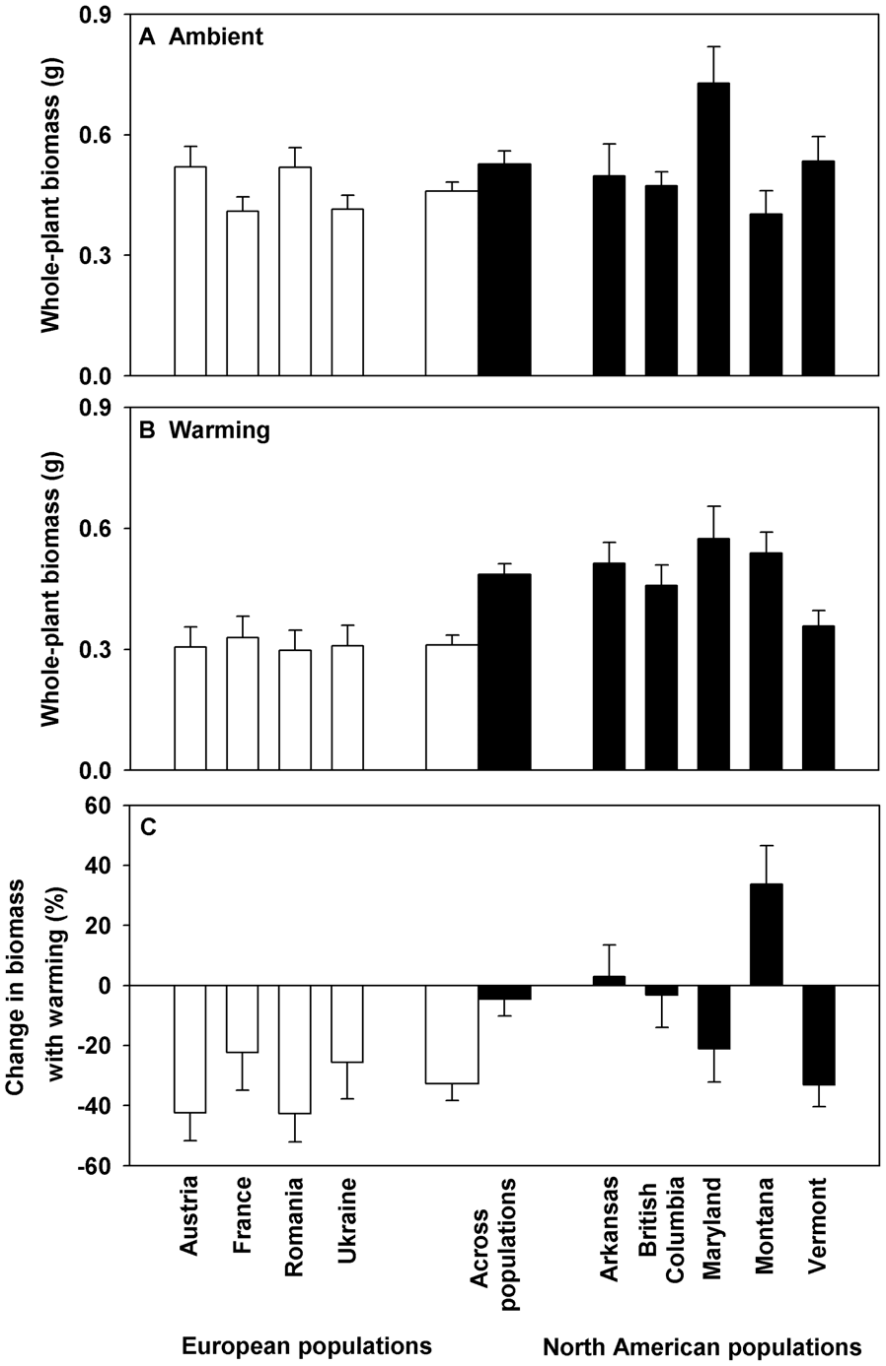
Whole-plant biomass of *Centaurea maculosa* populations from Europe and North America under either the ambient (A) or warming condition (B), and the change in whole-plant biomass for *C. maculosa* populations with warming (C). Means and 1 SE for each population are presented in the narrow bars, and means and 1 SE for each continent using the means of each population as replicates are presented in the two thicker bars in the center of the figure. See Table 1 for ANOVAs.

The mean biomass of *C. maculosa* populations across ambient and warming conditions was greater in North America (0.507±0.021 (1 SE) g) than in Europe (0.394±0.019 g) (Fig. 1A & B; Table 1, *F* = 16.842, *P*<0.000). Warming treatment decreased the biomass of *C. maculosa* across all populations from 0.503±0.021 g at the ambient to 0.408±0.020 g at the warmer condition (Fig. 1A & B; Table 1, *F* = 13.228, *P*<0.000). This decrease was primarily due to the decreased growth of European populations instead of North American populations (Fig. 1C). There was a significant interaction between continent and warming on the growth of *C. maculosa* (Table 1, *F* = 4.586, *P* = 0.034). For example, North American populations were 12% and 56% larger than European populations for the ambient (Fig. 1A) and the warming condition (Fig. 1B), respectively. Thus the difference in plant size between European and North American populations was enlarged with warming (Fig. 1C; Table 1, *F* = 12.463, *P*<0.000). There were significant differences in plant size among North American *C. maculosa* populations (Fig. 1; *P*<0.05), but not among European *C. maculosa* populations (Fig. 1; *P*>0.05).

**Table 1:** Analysis of variance (ANOVA) of the whole-plant biomass of individual plants, change in whole-plant biomass with warming, competitive effects, change in competitive effects with warming, competitive responses, and change in competitive responses with warming of *Centaurea maculosa* populations.

	Continent of population origin		Warming		Continent × warming	
	*F*_1,160/1,84_ *	*P*	*F*_1,160_	*P*	*F*_1,160_	*P*
Whole-plant biomass	16.842	**<0.000**	13.228	**<0.000**	4.586	**0.034**
Change in whole-plant biomass	12.463	**<0.000**				
Competitive effects	0.507	0.478	2.722	0.101	7.953	**0.005**
Change in competitive effects	16.534	**<0.000**				
Competitive response	0.545	0.462	47.029	**<0.000**	0.305	0.582
Change in competitive responses	0.402	0.528				

Values of *P*<0.05 are in bold.

*“1,160” is the degree of freedom for two-way ANOVAs, “1,84” is the degree of freedom for one-way ANOVAs.

**Figure 2:**
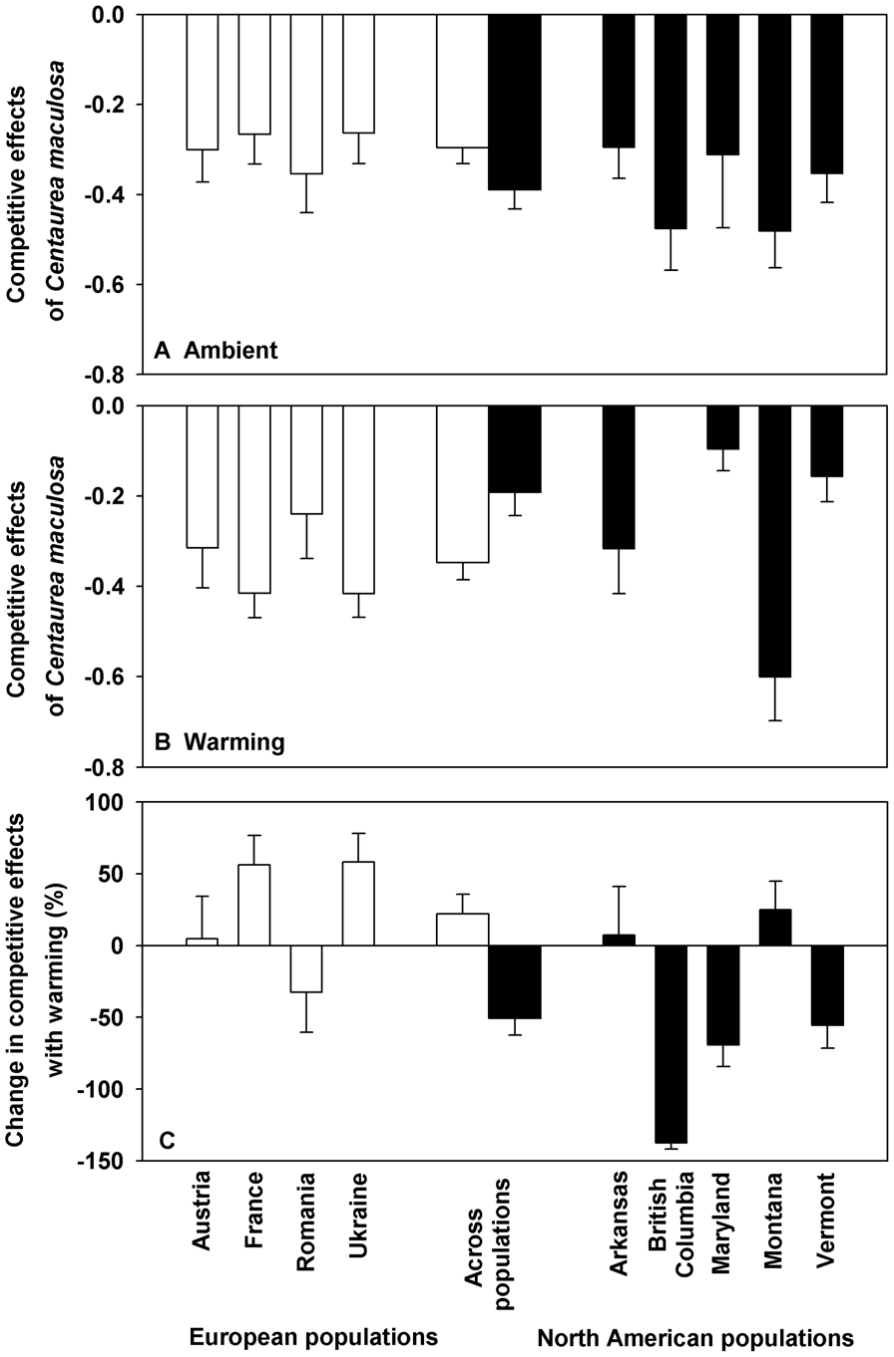
Competitive effects of *Centaurea maculosa* populations from Europe and North America under either the ambient (A) or warming condition (B), and the change in competitive effects for *C. maculosa* populations with warming (C). Means and 1 SE for each population are presented in the narrow bars, and means and 1 SE for each continent using the means of each population as replicates are presented in the two thicker bars in the center of the figure. See Table 1 for ANOVAs.

The RII values showed that the presence of *C. maculosa* exhibited inhibitory roles on the growth of *P. pratensis* (Fig. 2A & B). The continent (Table 1, *F* = 0.507, *P* = 0.478) and warming (Table 1, *F* = 2.722, *P* = 0.101) had no effects on the competitive effects of *C. maculosa* populations on *P. pratensis* (Fig. 2A & B). However, their interactions affected this competitive effect (Table 1, *F* = 7.953, *P* = 0.005). For example, the competitive effects of *C. maculosa* populations were smaller in Europe than in North America at the ambient (Fig. 2A); in contrast, the competitive effects of *C. maculosa* populations were greater in Europe than in North America at the warming condition (Fig. 2B). Warming treatment increased the competitive effect of European *C. maculosa* populations but decreased that of North American counterparts (Fig. 2C; Table 1, *F* = 16.534, *P*<0.000).

**Figure 3:**
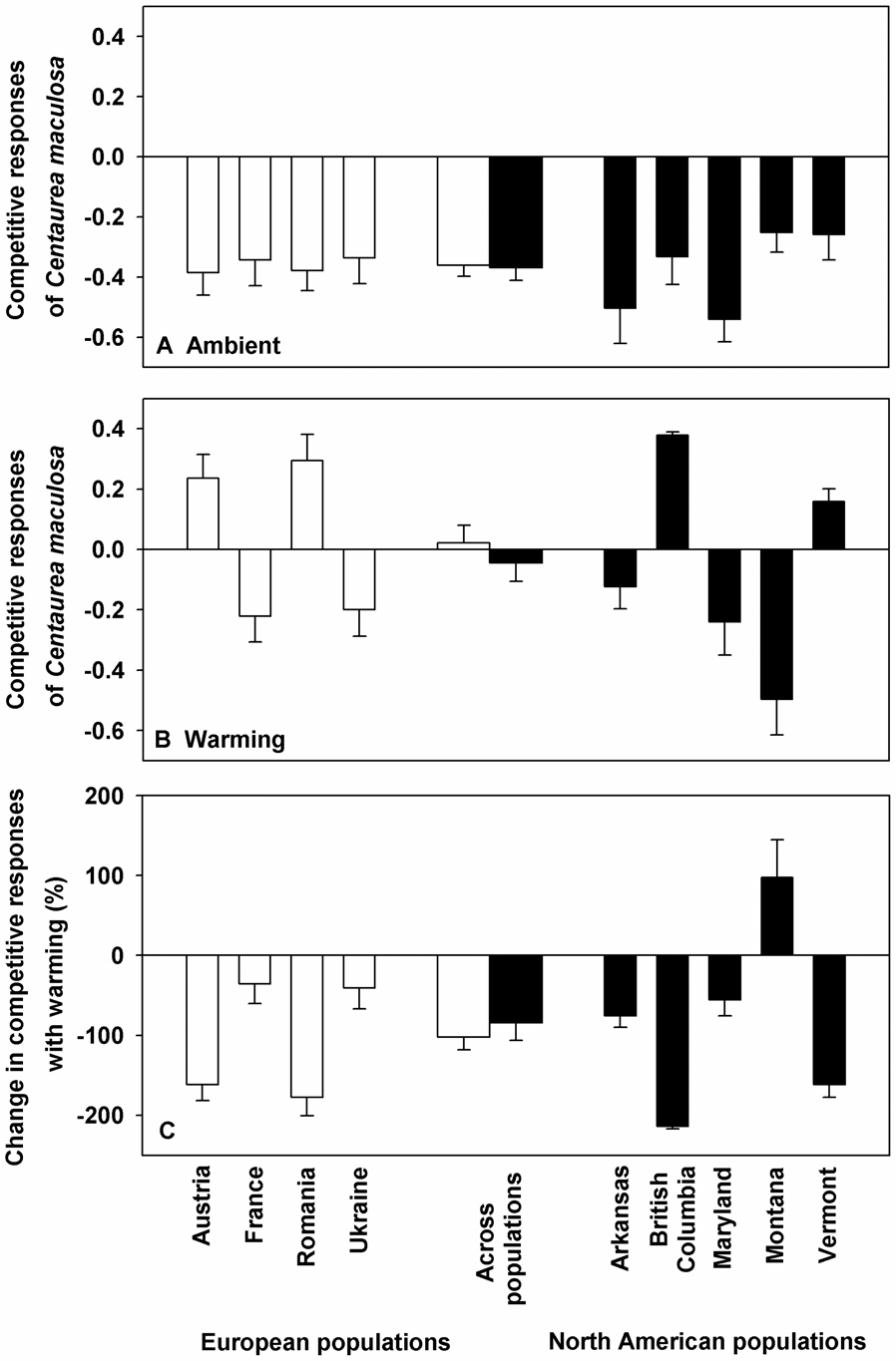
Competitive responses of *Centaurea maculosa* populations from Europe and North America under either the ambient (A) or warming condition (B), and the change in competitive responses for *C. maculosa* populations with warming (C). Means and 1 SE for each population are presented in the narrow bars, and means and 1 SE for each continent using the means of each population as replicates are presented in the two thicker bars in the center of the figure. See Table 1 for ANOVAs.

In the ambient condition, the presence of *P. pratensis* showed inhibitory effects on the growth of *C. maculosa* (Fig. 3A); in the warming condition, the presence of *P. pratensis* had either inhibitory effects or facilitative effects on the growth of *C. maculosa*, depending on population identity (Fig. 3B). Across two continents, warming treatment increased the competitive responses of *C. maculosa* populations to *P. pratensis* from −0.366±0.029 in the ambient to −0.015±0.043 in the warming condition (Fig. 3A & B; Table 1, *F* = 47.029, *P*<0.000). The continent of population origin (Table 1, *F* = 0.545, *P* = 0.462) and its interactions with warming (Table 1, *F* = 0.305, *P* = 0.582) had no effects on the competitive response. The differences in the competitive responses between European populations and North American populations did not vary with warming (Fig. 3C; Table 1, *F* = 0.402, *P* = 0.528).

**Figure 4:**
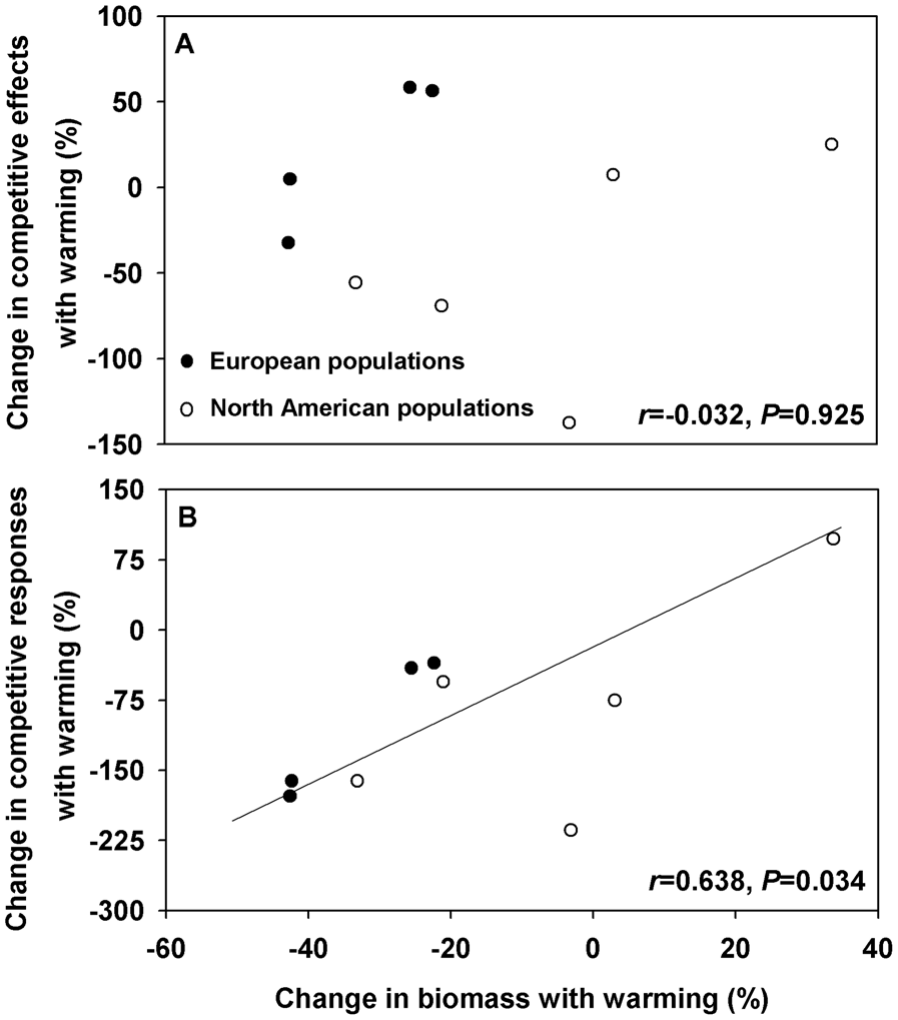
The relationship between the change in biomass with warming versus the change in competitive effects and competitive responses with warming for *Centaurea maculosa* populations from Europe and North America.

For all European and North American *C. maculosa* populations grown at the warming condition, the change in biomass was not associated with the change in competitive effects (Fig. 4A, *r* = −0.032, *P* = 0.925), but was positively correlated with the change in competitive responses (Fig. 4B, *r* = 0.638, *P* = 0.034). Thus, the changing plant size of *C. maculosa* grown alone with climate warming indicated its potential to avoid being suppressed by its neighbors but not its capacity to suppress its neighbors under the warming conditions.

## Discussion

This study is the first to our knowledge to examine the response of home and introduced populations to climate warming. *Centaurea maculosa* populations from home and introduced ranges did exhibit contrasting responses to climate warming. For example, North American *C. maculosa* populations were more tolerant to warming than European *C. maculosa* populations, but the former exhibited weaker competitive advantages than the latter under warmer climate. Thus these findings support our central hypothesis that identical warming differentially influences the growth and competitive ability of *C. maculosa* populations from its home and introduced ranges, and also add significantly to the consequences of climate warming for the risks of *C. maculosa* invasion.

In our study, herbivores were controlled as much as possible. Under the ambient condition, North American *C. maculosa* populations were larger and had a greater competitive ability than European counterparts, completely supporting the EICA hypothesis. However, in the warming condition North American *C. maculosa* populations were larger but had a smaller competitive ability than European counterparts, partially supporting the EICA hypothesis. The key prediction of the EICA hypothesis is that those plants with larger size have a higher competitive ability [20]. Our results suggest that warming can shift this positive correlation between growth and competitive ability, that is, the competitive ability of plants does not increase with increasing size in warmer climate. Thus the growth and competitive ability of invasive plants need to be considered simultaneously in related studies. For *C. maculosa*, rapid evolution of local populations within the introduced range is essential for its invasion success [7]–[9], but this evolution does not increase its invasiveness in the introduced range under climate warming.

Recent research suggests that warming can favor the growth of invasive plants [15], [16]. Under warming conditions, stimulation of growth is mainly due to altered reaction kinetics, acclimation to changing temperatures and water availability, and improved access to nutrients [16], [24]. In contrast, our findings suggest that climate warming can inhibit the growth of *C. maculosa*. This inhibitory effect resulting from warming can be attributed to stress effects in leaves via greater transpiration at higher temperatures and increased respiration [15], [16]. The responses of invasive plants to warming are also linked to soil water and nutrient availability. In our study, we provided adequate soil water and nutrients so that we can exclude the indirect negative effects of warming resulting from shortages of soil water and nutrients and the interactions of warming with these two factors. Overall, warming effects on growth are directly linked to source-sink relationships of photosynthesis; greater carbohydrate consumption by plant respiration during the previous night can stimulate photosynthesis in the following day in some cases, and the opposite is true in other cases [17], [18]. This different response of invasive plants to climate warming may also depend on invasive plant identity because different invaders exhibit contrasting invasive mechanisms. For example, the successful invasion of some exotics can be primarily ascribed to novel weapons [14], [25], and the successful invasion of some exotics can be mainly attributed to the release of enemies and increased competitive ability [20].

It is already known that climate warming is likely to alter the degree of competition amongst the co-occurring species and thus shifts community composition and structure [19]. Our findings show that climate warming can increase the competitive ability of *C. maculosa*. Specifically, warming decreased the competitive ability of *P. pratensis* against *C. maculosa*, but *C. maculosa* populations still maintained their high competitive ability in the warming condition. These findings demonstrate that warming-modulated competitive outcomes favor *C. maculosa* over *P. pratensis* and thus promote *C. maculosa* invasions. Since competitive outcomes between invasive and local species govern the success and patterns of invasive plants [26], [27], warming-induced shifts in competitive outcomes may enable home *C. maculosa* populations to be more invasive. Model simulations also suggest that climate warming tends to promote plant invasions through expanding suitable habitats [6], [28]. Thus increasing evidence shows that warming gives competitive advantages to exotic invaders [4], [5].

Our findings also demonstrate that the growth response of *C. maculosa* alone to warming cannot predict its ability to suppress other plants, but can predict its capacity to avoid being suppressed by its neighbors. In other words, if the plants of *C. maculosa* populations grow smaller under climate warming, then they may exhibit a greater capacity to tolerate competition; in contrast, those plants may have no greater ability to suppress other local plants because warming may allow their neighboring plants to witness relatively low decrease in growth.

It is important to note that all seeds of *P. pratensis* were collected from North America. Thus, *P. pratensis* may be an experienced competitor to North American *C. maculosa* populations and a naïve competitor to European *C. maculosa* populations. This phenomenon has been discussed in detail by Callaway & Aschehoug [25]. In our study this difference (i.e. experienced vs naïve competitor) may be weak because North American and European *C. maculosa* populations shared a similar response to the presence of *P. pratensis*, regardless of in the ambient or warming conditions. More importantly, North American *C. maculosa* populations exhibited stronger inhibitory effects on the growth of *P. pratensis* than European *C. maculosa* populations in the ambient conditions.

This study was a short-term (four months) experiment, but it provides substantial evidence that climate warming differentially affects the growth and competitive ability of *C. maculosa* populations from its home range (Europe) and introduced range (North America). Our findings provide an indication that climate warming may help *C. maculosa* populations be more invasive from its home range but not introduced range, and also suggest that climate warming may drive *C. maculosa* invasion through increasing competitive advantages. In the field, *C. maculosa* plants commonly grow with diverse local plant species with various ages so that its risks depend on other local plant species. It is required to further ascertain whether climate warming can effectively tip the balance between *C. maculosa* and its multiple local species in the field.
